# Lottery incentives have short‐term impact on ART initiation among men: results from a randomized pilot study

**DOI:** 10.1002/jia2.25519

**Published:** 2020-06-26

**Authors:** Ruanne V Barnabas, Alastair van Heerden, Margaret McConnell, Adam A Szpiro, Meighan L Krows, Torin T Schaafsma, Thulani Ngubane, Rose B Nxele, Philip Joseph, Jared M Baeten, Connie L Celum, Heidi van Rooyen

**Affiliations:** ^1^ Department of Global Health and Division of Allergy and Infectious Diseases University of Washington Seattle WA USA; ^2^ Vaccine and Infectious Diseases Division Fred Hutchinson Cancer Research Center Seattle WA USA; ^3^ Human Sciences Research Council Sweetwaters KwaZulu‐Natal South Africa; ^4^ MRC/Wits Developmental Pathways for Health Research Unit University of the Witwatersrand Johannesburg‐Braamfontein South Africa; ^5^ Harvard T. H. Chan School of Public Health Boston MA USA; ^6^ Department of Biostatistics University of Washington Seattle WA USA

**Keywords:** conditional incentives, HIV, men, South Africa, lottery, ART, continuum of care

## Abstract

**Introduction:**

Among people living with HIV in South Africa, viral suppression is lower among men than women. The study aim was to test the impact of lottery incentives, which reward positive health choice (e.g. antiretroviral therapy (ART) linkage) with a chance to win a prize, on strengthening the HIV care continuum including ART initiation and viral suppression for men.

**Methods:**

We conducted a randomized, prospective trial of lottery incentives in the context of HIV testing and linkage to ART in rural KwaZulu‐Natal, South Africa. Men living with HIV were randomly allocated to: lottery incentives and motivational text messages or motivational text messages only. Lottery prize eligibility was conditional on clinic registration, ART initiation, or viral suppression by one, three and six months respectively. After completing each continuum step, participants in the lottery group were notified whether they had won and were encouraged to continue in care. Lottery prizes were either a mobile phone, data or a gift card (valued at R1000/$100). Kaplan–Meier curves were plotted to determine time to ART initiation by study group. The primary outcome was viral suppression at six months.

**Results:**

Between November 2017 and December 2018, we tested 740 men for HIV and enrolled 131 HIV‐positive men who reported not being on ART. At baseline, 100 (76%) participants were 30 years and older, 95 (73%) were unemployed and the median CD4 count was 472 cells/μL. At study exit, 84% (110/131) of participants had visited a clinic and 62% (81/131) were virally suppressed. Compared to motivational text messages, lottery incentives decreased the median time to ART initiation from 126 to 66 days (*p* = 0.0043, age‐adjusted Cox regression) among all participants, and, from 134 days to 20 days (*p* = 0.0077) among participants who were not virally suppressed at baseline. Lottery incentives had an inconclusive effect on clinic registration (RR = 1.21, 95% CI: 0.83 to 1.76) and on viral suppression at six months (RR = 1.13, 95% CI: 0.73 to 1.75) compared to motivational text messages.

**Conclusions:**

Conditional lottery incentives shortened the time to ART initiation among South African men. Behavioural economics strategies strengthen linkage to ART, but the study power was limited to see an impact on viral suppression.

**Clinical Trial Number:**

NCT03808194.

## 1 INTRODUCTION

Of the 4.5 million South Africans on antiretroviral therapy (ART) only a third are men, despite men making up 45% of people living with HIV nationally [1]. Viral suppression is lower among men (47%) than women (58%) [1]. In addition, men living with HIV are underrepresented throughout the HIV care continuum, being less likely to test, link to care, initiate ART and more likely to be lost to follow‐up compared to women living with HIV [2, 3, 4, 5]. In a previous study of an optimized testing and linkage to care package of community‐based HIV testing, referral, text message reminders and lay‐counsellor support, we were only able to achieve 60% linkage to HIV care and ART among men [6]. Men living with HIV who are not virally suppressed are at risk for HIV‐associated morbidity and mortality, and their HIV‐negative partners are at risk of HIV acquisition. Thus, innovative strategies are needed to motivate HIV‐positive men to engage in care, and specifically to initiate and adhere to ART.

Conditional lottery incentives, which reward positive behaviour choices with a chance to win a prize, are a behavioural economics approach to motivate present‐day behaviour for future health gains (e.g. engagement in HIV care to increase long‐term life expectancy). Men tend to have preferences that are more risk‐tolerant, which is described in health economic literature [7]. As a result, men may be willing to take on greater risks in seeking greater rewards. Also, in the context of accessing healthcare, men face competing risks and may choose the benefit of informal employment over engagement in HIV care. Lottery strategies are hypothesized to benefit men because men tend to have preferences that are more risk‐tolerant [7]. Furthermore, lottery incentive strategies have been successfully used to increase uptake of HIV prevention and treatment [8, 9, 10, 11, 12]; in one example, lottery incentives, conditioned on being Sexually Transmitted Infection (STI) negative, decreased HIV incidence by 60% among individuals who scored high on risk questionnaires in Lesotho [9, 13], demonstrating one of the largest effects to date of a behavioural intervention for HIV prevention.

Given the success of lotteries to engage men and risk‐takers in HIV prevention, we hypothesized that lottery incentives have the potential to overcome both structural and behavioural factors for linking HIV‐positive men to care, addressing logistical challenges and risk preferences specific to men [4]. Also, while lottery incentives have shown short‐term impact, it is not known if the effect is sustained over time and whether lottery incentives would support sustained viral suppression in addition to ART initiation. Therefore, we tested the effectiveness of lottery incentive strategies on time to ART initiation and the proportion of men achieving viral suppression over time in KwaZulu‐Natal, South Africa (the Lotto to Link Study). We tested both the short term, ART initiation, and long term, viral suppression, effects of lottery incentives.

## 2 METHODS

### 2.1 Study design and participants

We conducted an individual randomized study of conditional lottery incentives to link men living with HIV to care, ART initiation and viral suppression over time. The study was conducted from the Human Sciences Research Council’s Sweetwaters field office located in the Greater Edendale area, KwaZulu‐Natal, South Africa. The Greater Edendale area is characterized by very high HIV prevalence – 30% prevalence – high unemployment and low per capita income (under USD $2 per day) [14]. The study was supported by local department of health staff and conducted at the Sinathing Clinic, which provides HIV prevention and treatment according to national South African guidelines [15]. HIV care and treatment, including ART and laboratory tests, are provided at a nominal cost or free of charge at public clinics. The study staff worked closely with clinic staff to facilitate study recruitment and successful execution of study procedures.

Eligible participants were age 18 years or older, identified as male gender, resident in the study community for the duration of follow‐up, able and willing to provide informed consent, had a positive test for HIV using the national rapid HIV antibody testing algorithm, not currently on ART, and had access to confidential text messaging. Men living with HIV were eligible, regardless of whether they were newly diagnosed, if they were not currently taking ART.

All participants provided written informed consent. The University of Washington Institutional Review Board and the Human Sciences Research Council Research Ethics Committee approved this study.

### 2.2 Randomization and masking

Participants were randomized 1:1 to either conditional lottery incentives and motivational text message support for linking to care or motivational text messages only (control group). The unit of randomization was the individual. The randomization sequence was predetermined and available through the staff mobile phone app for each participant enrolled. The randomization allocation was not revealed to staff or the participant until all screening procedures were completed and eligibility was confirmed. The randomization code was generated at the University of Washington (UW) International Clinical Research Center (ICRC). The random allocation was programmed into the mobile phone app by Mobenzi Researcher (Durban, South Africa), with UW ICRC oversight. Due to the difficulty of blinding the study team and study participants to the intervention, the study was unblinded; however, all participants receive two‐way, supportive motivational text messages. The laboratory staff, who assessed the primary outcome of plasma HIV viral load, were blinded to the allocation of participants as were the study investigators.

### 2.3 Procedures

We conducted community sensitization through community events and engaged with local stakeholders including local community leaders and department of health officials. We met with community members and discussed the study rationale and answered questions. Once local community and department of health permissions were obtained, recruitment for the study began.

Participants were recruited for screening through community‐based HIV testing and counselling (HTC) at home, through mobile HTC (testing from mobile vans), and by referral of newly identified men living with HIV and not yet on ART from clinics. Men and women were offered HIV testing, but only men were eligible for the intervention. Comprehensive counselling, including disclosure counselling for couples, on HIV treatment and prevention was provided. Persons who tested positive for HIV but who were not eligible for the study, were referred to local clinics for care. Persons who tested HIV negative were referred for prevention services. Additional health services, specifically measurement of blood pressure for hypertension screening, were offered as services to increase HIV testing uptake. Participants were referred for additional clinical services following local guidelines.

Participants completed a demographic questionnaire including their education level attained, employment status, risk behaviour and previous HIV testing and care. Participants completed a hypothetical lottery questionnaire in which they could choose between a fixed sum of money and a prespecified chance of winning another sum of money. The hypothetical gambling questionnaire assessed their willingness to risk a small guaranteed reward for the chance of a large reward [16]. The results of these gambling questions form the basis of assessing the risk‐tolerance score [9]. We used a fingerprint biometric, which was translated to a binary code, to identify the participant at subsequent visits.

At baseline we collected dried blood spot (DBS) cards to assess HIV viral load at enrolment. Detectable viral load was not confirmed prior to study enrolment; participants reported that they were not engaged in care. Thus, this study population likely represents men living with HIV who were re‐engaging in care and men initiating ART for the first time. To assess HIV stage and eligibility for opportunistic infection prophylaxis, point‐of‐care CD4 testing was conducted. Participants were counselled about HIV natural history, the benefits of viral suppression, ART safety and efficacy, and had the opportunity to ask questions. Participants were referred to the Sinathing Clinic for HIV care and treatment, the nearest clinic to the community where testing was conducted. At the end of the screening visit, study staff ensured that questions were answered and participants understood their results.

Eligible participants were enrolled and randomized to either the conditional lottery incentive group plus motivational text messages (SMS + Lottery) or motivational text messages (SMS) only. Participants in both groups received an optimized ART linkage package, including a clinic referral card and two‐way text messages to support linkage to ART. At months 1, 2 and 3, participants received a neutral, encouraging text message, for example “Make good decisions for your health today!,” with a number to text if they needed additional help. Upon arrival at the clinic, participants provided their fingerprint as identification and confirmation of study participation. The study team member, based at the clinic, recorded the purpose of the visit, that is clinic registration, ART initiation, ART refill or Other.

In addition, participants in the lottery group received an immediate text message after visiting the clinic, which indicated that they had been entered into the lottery for completing the next step in the HIV care continuum. One‐week later participants received a text message indicating that they had won the lottery or not and encouraging them to continue to link to care for additional opportunities to win the lottery. The lottery winners were predetermined through a random draw prior to the study start, allowing almost real time notification of winners once they completed each conditional step. The minimum probability of winning the lottery was 1 in 56 at each stage (clinic linkage, ART initiation and viral suppression) – this probability was shared with participants at enrolment. If winning participants did not meet the lottery conditions, that is had not linked to care, initiated ART, or achieved viral suppression which we confirmed with the clinic, the incentive was returned to the pool and a new winner selected.

Lottery eligibility was conditional on clinic registration, ART initiation and viral suppression by one, three and six months respectively. Lottery prizes were either a mobile phone, data or a gift card (all valued at R1000/$100). Participants were eligible for the lottery at each of the steps in the HIV continuum, regardless of whether they had won previously. The lottery prize was deemed sufficient to encourage linkage to care through community discussions. Once a lottery prize had been won, general information was provided to participants in the lottery group that someone had won the lottery prize and sharing the details of the prize selected.

At the six‐month exit visit all participants completed a questionnaire on uptake of HIV care, clinic visits, ART initiation, ART adherence barriers to care, acceptability and durability of lottery incentive strategies and risk behaviour. Study staff reviewed the clinic records to confirm the medication and dates of visits. We collected plasma for HIV viral load testing, and provided the result to participants to support their HIV care.

### 2.4 Outcomes

The prespecified primary outcomes were linkage to the ART clinic, ART initiation and viral suppression [defined as viral load below the assay limit of detection (<20 copies/mL)] at six months among the intention‐to‐treat population. Linkage to the ART clinic and ART initiation were assessed by study staff recording the reason for the clinic visit and verification in the clinic chart. Prespecified secondary outcomes included time to ART initiation and evaluation of the primary outcomes among individuals who were classified as risk‐tolerant using the risk score.

### 2.5 Statistical analysis

We estimated that a sample size of 120 eligible participants would be needed to have at least 80% power to see an absolute 25% difference or more in viral suppression in the lottery group vs. the motivational text message group. Based on our previous work, we estimated that viral suppression would be 60% in the control group [6]. With a 5% loss to follow‐up, we expected to retain 57 per group.

Relative risks (RR) of viral suppression, ART initiation and clinic registration and 95% robust confidence intervals were calculated comparing randomization groups using a log‐linear regression, assuming a working independence Poisson model (i.e. generalized estimating equations). All regressions included adjustment for age (≥30 years). Hypothesis tests for RR ≠ 1 were based on two‐sided Wald *p*‐values < 0.05. The number and percentage of participants who initiated ART and the median time to ART initiation was plotted using a Kaplan–Meier curve by study group to illustrate the rate at which participants initiated ART, preferentially using the date of dispensation abstracted from the clinic chart if different from the date reported by the participant. Based on previous studies [6] we expected some participants to enrol with a suppressed viral load, despite reporting not currently being on ART. Therefore, we assessed the impact of the lottery incentive among persons who had detectable viral load at baseline to explore the intervention’s effect on participants presumably not already on treatment, in a modified‐intension‐to‐treat analysis.

As described by Nyquist and colleagues [9], we constructed an indicator variable “risk‐loving”/risk‐tolerant on a scale of 0.0 to 1.0, where 1.0 is a risk‐tolerant and 0.0 is a risk‐averse participant. Participants who chose the lottery even when the fixed amount offered is equal or greater than 500 ZAR (50% chance of winning 1000 ZAR) were assigned a risk‐tolerant score of 1.0 and those who consistently chose the fixed amount below the expected value of the lottery (500 ZAR) received a risk‐tolerant score of 0.0 (i.e. they are risk‐averse). To generate the “risk‐tolerance index” (RTI), responses were normalized from 0 (safest) to 100 (riskiest) for the hypothetical lotteries in the questionnaire, and the mean of the lotteries were combined for the RTI. To explore a pragmatic definition of risk, we defined risk as having a detectable viral load as a marker of seeking care late. We used R 3.5 for all the analyses.

### 2.6 Laboratory analysis

Community HIV testing was conducted using blood obtained by finger‐stick and tested using rapid serologic tests according to national guidelines by ABON HIV Rapid test (Alere, Waltham, MA, USA), and First Response HIV Test (Premier Medical Corporation Ltd, Watchung, NJ, USA) for confirmation, with HIV 1/2 Gold Screening Test (G‐Ocean, Singapore) as a tie breaker when needed. Point‐of‐care CD4 testing (Alere, PIMA™, Waltham, MA, USA) was conducted in the home or mobile van using a finger‐stick specimen. Plasma and DBS card were obtained and transported to the reference laboratory for HIV viral load testing by polymerase chain reaction (bioMérieux, Craponne, France) with a limit of detection of 20 copies/mL.

## 3 RESULTS

**Figure 1:**
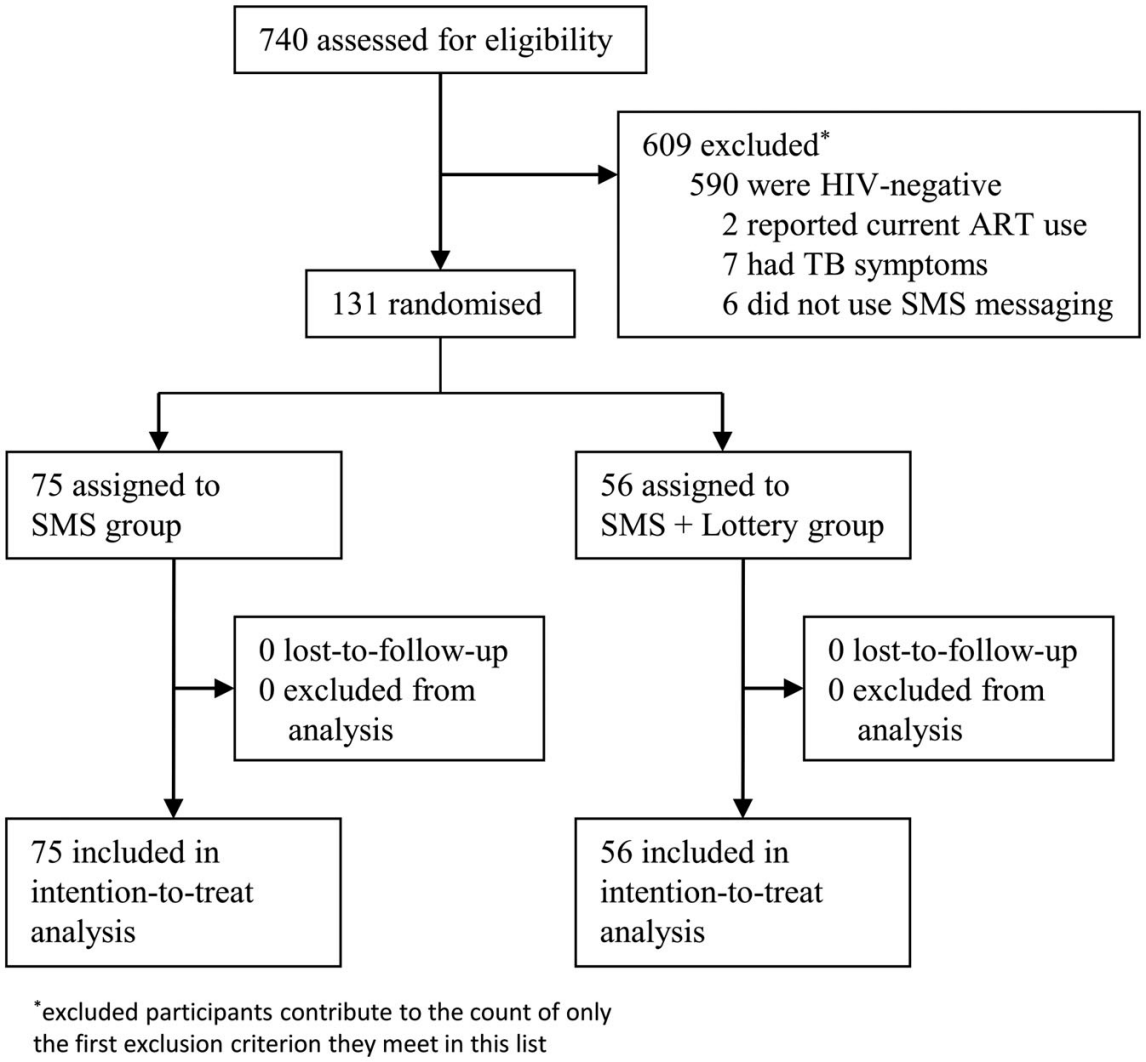
Study profile. ART, antiretroviral therapy; TB, tuberculosis.

Between November 2017 and December 2018, we tested 740 men for HIV through community HCT and clinic referral and 150 (20%) tested HIV positive. Of the 740 men tested, 609 were excluded; 590 tested HIV negative, 19 did not meet other inclusion criteria. No participants declined study participation. Of the 131 eligible men enrolled in the study, 56 were randomly assigned to the lottery plus text message group and 75 were assigned to the text message only group (Figure 1). The randomization was not blocked and while the group sizes are not even, the difference was not statistically significant and baseline characteristics that could be potential confounders were even by group. No participants were lost‐to‐follow‐up. The primary analysis includes 100% of participants enrolled.

At baseline, 100 (76%) participants were 30 years and older, 113 (86%) attained secondary education level, 95 (73%) were unemployed, 90 (69%) were single, 85 (65%) reported one current sex partner and 102 (78%) reported no condom use at last sex, which was comparable between the study groups (Table 1). Clinical characteristics were also similar between the groups with a median CD4 count of 472 cells/μL. All participants reported that they were currently not taking ART, but surprisingly 73 (56%) percent of participants were suppressed at baseline with a viral load of <20 copies/mL. The median follow‐up time was 8.8 months. All three lottery prizes were collected by participants.

**Table 1:** Baseline characteristics

	Total	SMS	SMS + Lottery
	(n = 131)	(n = 75)	(n = 56)
Age			
18 to 24	15 (11%)	8 (11%)	7 (12%)
25 to 29	16 (12%)	9 (12%)	7 (12%)
30 to 49	90 (69%)	50 (67%)	40 (71%)
≥50	10 (8%)	8 (11%)	2 (4%)
Education level			
Primary	18 (14%)	11 (15%)	7 (12%)
Secondary	106 (81%)	58 (77%)	48 (86%)
Tertiary and above	7 (5%)	6 (8%)	1 (2%)
Occupation			
Unemployed	95 (73%)	52 (69%)	43 (77%)
Labourer/semi‐skilled/other	29 (22%)	19 (25%)	10 (18%)
Trade/sales	4 (3%)	3 (4%)	1 (2%)
Student	3 (2%)	1 (1%)	2 (4%)
Marital status			
Married	6 (5%)	1 (1%)	5 (9%)
Living together, not married	3 (2%)	2 (3%)	1 (2%)
In a relationship, not married	32 (24%)	24 (32%)	8 (14%)
Single	90 (69%)	48 (64%)	42 (75%)
Number of current sex partners			
0	8/130 (6%)	5/74 (7%)	3 (5%)
1	85/130 (65%)	47/74 (64%)	38 (68%)
≥2	37/130 (28%)	22/74 (30%)	15 (27%)
Condom used at last sex	28/130 (22%)	13/74 (18%)	15 (27%)
Baseline CD4 count^a^ (POC, cells/mL)			
<100	2/74 (3%)	2/42 (5%)	0/32 (0%)
100 to 349	24/74 (32%)	10/42 (24%)	14/32 (44%)
350 to 499	14/74 (19%)	7/42 (17%)	7/32 (22%)
≥500	34/74 (46%)	23/42 (55%)	11/32 (34%)
Baseline viral load (DBS, copies/mL)			
<20	73 (56%)	42 (56%)	31 (55%)
20 to 999	15 (11%)	8 (11%)	7 (12%)
1000 to 9999	18 (14%)	10 (13%)	8 (14%)
≥10,000	25 (19%)	15 (20%)	10 (18%)

^a^ Functioning point‐of‐care CD4 count machines were not available for the entire study, thus only 74 CD4 count measures are provided.

In the primary intention‐to‐treat analyses, at six months, registration at the clinic was high in both groups; 77% in the SMS only group and 93% in the lottery plus SMS group, which were not statistically different (adjusted relative risk [aRR] 1.21, 95% CI 0.83 to 1.76) (Table 2). The proportion of participants initiating ART by six months was high in both groups; 76% in the text message group and 93% in the lottery plus SMS group; which were not statistically different between the groups (aRR 1.23, 95% CI 0.84 to 1.79). There was no difference in viral suppression at six months; 59% of participants in the SMS group and 66% in the lottery group had a viral load of <20 copies/mL (aRR 1.13, 95% CI 0.73 to 1.75).

**Table 2:** Effect of conditional lottery incentives on clinic registration, ART initiation and viral suppression at six months

	Registered at clinic		Initiated ART		Viral load <20 copies/mL	
	n (%)	aRR^a^ (95% CI)	n (%)	aRR^a^ (95% CI)	n (%)	aRR^a^ (95% CI)
SMS	58/75 (77%)	Reference	57/75 (76%)	Reference	44/75 (59%)	Reference
SMS + Lottery	52/56 (93%)	1.21 (0.83 to 1.76)	52/56 (93%)	1.23 (0.84 to 1.79)	37/56 (66%)	1.13 (0.73 to 1.75)

^a^ aRR, adjusted relative risk. All analyses are adjusted for age.

**Figure 2:**
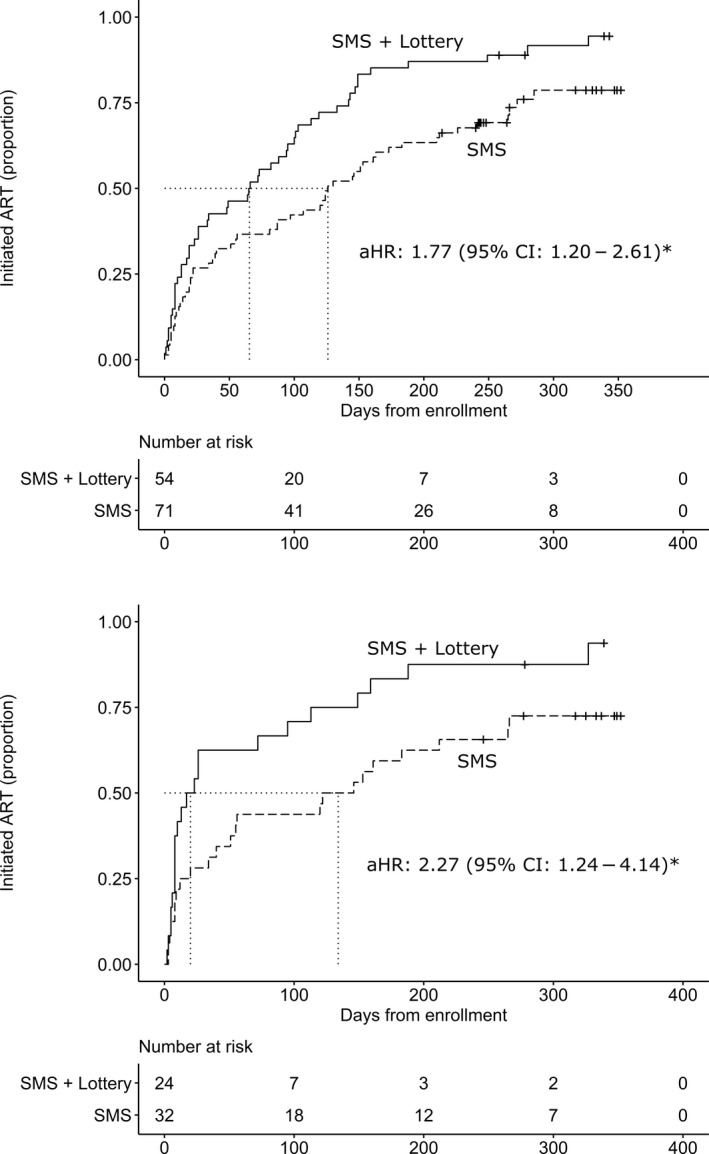
Probability of **(a)** ART initiation for all participants receiving; **(b)** ART initiation for participants with detectable viral load at baseline receiving (1) SMS + Lottery incentive group or (2) SMS only group. *Adjusted for age less than 30 years.

Compared to motivational text messages alone, lottery incentives decreased the median time to ART initiation to 66 from 126 days (adjust hazard ratio (aHR) 1.77, 95% CI 1.20 to 2.61, *p* = 0.0043) among all participants (Figure 2a), and to 20 days from 134 days (aHR 2.27, 95% CI 1.24 to 4.14 *p* = 0.0077) among participants who had detectable viral load at baseline (Figure 2b).

Thirty‐four participants in the SMS group and 30 in the SMS plus lottery group were assessed as being risk‐tolerant based on their responses to hypothetical gambling questionnaires. There was no statistically significant difference in the proportion registering at the clinic, initiating ART, or achieving viral suppression by study group (Table 3). In planned modified intention‐to‐treat analysis, men with detectable viral load at baseline were about a quarter to a third more likely to register at the clinic (aHR 1.25, 95% 0.71 to 2.22), initiate ART (aHR 1.30, 95% CI 0.73 to 2.32) and become virally suppressed (aHR 1.35, 95% 0.60 to 3.06), although the small sample size did not reach statistical significance (Table 3).

**Table 3:** Effect of conditional lottery incentives on clinic registration, ART initiation and viral suppression at six months among persons with high‐risk tolerance scores and persons with detectable viral load at baseline

	Registered at clinic		Initiated ART		Viral load <20 copies/mL	
	n (%)	aRR^a^ (95% CI)	n (%)	aRR^a^ (95% CI)	n (%)	aRR^a^ (95% CI)
Risk‐tolerant						
SMS	27/34 (79%)	Reference	26/34 (77%)	Reference	20/34 (59%)	Reference
SMS + Lottery	28/30 (93%)	1.22 (0.72 to 2.09)	28/30 (93%)	1.27 (0.74 to 2.18)	20/30 (67%)	1.16 (0.62 to 2.18)
Detectable baseline viral load						
SMS	25/33 (76%)	Reference	24/33 (73%)	Reference	12/33 (36%)	Reference
SMS + Lottery	23/25 (92%)	1.25 (0.71 to 2.22)	23/25 (92%)	1.30 (0.73 to 2.32)	12/25 (48%)	1.35 (0.60 to 3.06)

^a^ aRR, adjusted relative risk. All analyses are adjusted for age less than 30 years.

## 4 DISCUSSION

In this pilot randomized trial of conditional incentives to strengthen the continuum of HIV care, particularly among men living with HIV, lottery incentives decreased the time to ART initiation overall, particularly among persons with detectable viral load. Clinic registration, ART initiation and viral suppression were higher in the lottery and motivational text message arm, but this did not reach statistical significance in this pilot study. Furthermore, effects were seen early in the continuum of care, that is ART initiation, but were smaller in magnitude later in the continuum, that is viral suppression. The results were not different by risk‐tolerance, although men with detectable viral load at enrolment, who were potentially taking greater risks with their health, initiated ART more quickly, half initiating within three weeks in the lottery incentive group compared to 19 weeks in the control group.

While incentives did not have a significant effect on ART uptake, the loss‐to‐follow‐up after six months was notable. While more than 90% of participants in the lottery incentive group registered at the clinic and initiated ART, only two‐thirds were virally suppressed – a loss of a third of clients. This suggests that the behavioural economics approach of conditional incentives may have a short‐term impact on behaviour and likely requires additional strategies to sustain engagement in care. Well‐timed cues for new habit formation, such as lottery incentives, could be added to ART programmes to increase daily ART adherence [17]. The initial “pull” of lottery incentives may increase engagement in care, as manifested by high clinic registration and ART initiation rates, but over time other concerns such as logistics of visiting the clinic and stigma appear to outweigh the incentive effect. Also notable is the proportion of men virally suppressed at baseline (56%), who reported that they were not currently on ART, which may have been motivated by receiving more supportive care through the study. With the remaining sample size of 33 participants in the control group and 25 in the intervention group, our power to see an impact on viral suppression was reduced. Finally, while we did not assess the impact of motivational two‐way text messages, this type of intervention has demonstrated utility in other studies [18].

One potential explanation for the findings might be related to the timing of the intervention. We saw success when the intervention occurred after taking the step of engagement – that is fewer days to register at the clinic and initiate ART when patients received a reminder, an immediate text message confirming their lottery entry, and one week later they received the result of the lottery. However, for adherence to and persistence with daily ART pill‐taking, additional strategies may be needed. Conditional incentives may be appropriate for a specific, one‐time behaviour, but fatigue may prevent the persistence required for daily pill taking.

Among men with detectable viral load, the lottery intervention appeared to increase viral suppression by more than a third, but the small sample size limited the power of this analysis to reach statistical significance. This suggests that a “one‐size‐fits‐all” approach to engagement in the HIV care continuum may not maximize the impact of strategies such as lottery incentives, which could be reserved for persons with detectable viral load as a marker of risk‐tolerance but this would need to be tested to ensure that this did not create an incentive to stop taking ART. Behavioural economic approaches require testing among priority populations to increase access to HIV care.

Our findings were consistent with other incentive studies which found a modest or no effect of incentives on sustained viral suppression [12, 19, 20, 21]; a previous study also had a significant proportion of participants with viral suppression at baseline [20]. However, incentives can work to increase overall viral suppression in specific settings where suppression through usual care is low [11]. An important question is why viral suppression was 62% at the end of six months, below the UNAIDS target of 73% [22]. One explanation is that while lottery incentives provide short‐term motivation, barriers to clinic‐based care such as transportation, logistics and clinic hours decrease linkage overall [23]. This suggests that other interventions, such as delivery interventions to overcome logistic barriers to clinic access, and simplified regimens such as long‐acting injectable ART, which overcome the need for daily adherence, need to be part of a package to engage and retain men in care.

Our study had several limitations. First, although men reported that they were not currently on ART, more than half were virally suppressed at baseline. The impact of viral suppression at baseline is that the study population is likely a combination of men living with HIV engaging in care for the first time, men re‐engaging in care and men seeking care through an alternate method. Thus, the impact of lottery incentives for men living with HIV with a detectable viral low (i.e. not engaged in care) was assessed among a subset of 58 men and the analysis was underpowered to show an effect of 25% increase in viral suppression. This observation of undisclosed ART use has been described in previous studies [24, 25] and may be due to high demand for services among men. To be eligible for the study, participants reported that they were not in care. The higher than expected proportion of participants suppressed at baseline would affect the control and treatment group equally, so the study findings would still be valid even if they might underestimate the potential effects. However, because South African men are a priority group to reach for HIV care, data on the impact of conditional incentives in strengthening linkages to care contributes to a package of services for ART initiation and retention; specifically, the effect among persons with a detectable viral load at baseline. For these persons, while underpowered, the results were encouraging for lottery incentives which could be further investigated. Using point‐of‐care viral load testing to establish whether persons living with HIV have a detectable viral load may help direct the use of incentive strategies to persons who would benefit from the behavioural “nudge.” Second, while we were able to link the clinic registration and ART initiation lottery to those visits, we were not able to do so as easily with the viral load results due to limited access to the laboratory results system. Ideally, being suppressed would trigger a prompt lottery entry message and a detectable viral load would trigger additional support and the prospect of future lottery entry. Lastly, since linkage to care and ART initiation were slow overall, longer windows for linkage, with additional opportunities for incentives, might have increased the proportion achieving viral suppression.

## 5 CONCLUSIONS

In summary, lottery incentives for men at each stage of the HIV care continuum decreased time to ART initiation with possible increases in clinic registration and ART initiation compared to the control group of motivational text messages. It is possible that this intervention may be best suited to men with detectable viral loads, for whom the intervention had the biggest impact shortening the time to ART initiation. However, it is likely that men will require additional services to sustain retention in care over time, particularly services aimed at simplifying delivery and increasing adherence.

## COMPETING INTERESTS

We declare that we have no conflicts of interests.

## AUTHORS’ CONTRIBUTIONS

RVB, AvH, JMB, CC and HvR designed the study. AvH, MK, RBN, PJ and TN oversaw the implementation of the study. RVB wrote the first draft of the paper, which was revised by all authors. All authors contributed to design and execution of the study, as well as to the interpretation of findings. MM provided expertise on behavioural economics. TS did the statistical analysis with input from AAS, RVB, AvH, HvR and CC. All the authors approved the final version of the paper for submission.
